# Puerarin Alleviates Neuropathic Pain by Inhibiting Neuroinflammation in Spinal Cord

**DOI:** 10.1155/2014/485927

**Published:** 2014-06-24

**Authors:** Ming Liu, Kaijun Liao, Changxi Yu, Xuejun Li, Suhuan Liu, Shuyu Yang

**Affiliations:** ^1^Xiamen Diabetes Institute, The First Affiliated Hospital of Xiamen University, 55 Zhenhai Road, Xiamen 361003, China; ^2^Huazhong University of Science & Technology, China; ^3^Department of Pharmacology, College of Pharmacy, Fujian Medical University, China; ^4^Division of Endocrinology and Diabetes, The First Affiliated Hospital of Xiamen University, China; ^5^Central Laboratory, The First Affiliated Hospital of Xiamen University, China

## Abstract

Neuropathic pain responds poorly to drug treatments, and partial relief is achieved in only about half of the patients. Puerarin, the main constituent of *Puerariae Lobatae Radix*, has been used extensively in China to treat hypertension and tumor. The current study examined the effects of puerarin on neuropathic pain using two most commonly used animal models: chronic constriction injury (CCI) and diabetic neuropathy. We found that consecutive intrathecal administration of puerarin (4–100 nM) for 7 days inhibited the mechanical and thermal nociceptive response induced by CCI and diabetes without interfering with the normal pain response. Meanwhile, in both models puerarin inhibited the activation of microglia and astroglia in the spinal dorsal horn. Puerarin also reduced the upregulated levels of nuclear factor-*κ*B (NF-*κ*B) and other proinflammatory cytokines, such as IL-6, IL-1*β*, and TNF-*α*, in the spinal cord. In summary, puerarin alleviated CCI- and diabetes-induced neuropathic pain, and its effectiveness might be due to the inhibition of neuroinflammation in the spinal cord. The anti-inflammation effect of puerarin might be related to the suppression of spinal NF-*κ*B activation and/or cytokines upregulation. We conclude that puerarin has a significant effect on alleviating neuropathic pain and thus may serve as a therapeutic approach for neuropathic pain.

## 1. Introduction

Neuropathic pain is caused by aberrant sensory processing in either the peripheral- and/or the central nervous system (CNS) and can be induced by a variety of factors, including traumatic damage, infection, and diabetes [1]. Abnormal neuronal activity plays a fundamental role in the pathogenesis of neuropathic pain, while current treatments that suppress aberrant neuronal activity generally lack efficacy, in addition to their many undesirable side effects [2]. Recent studies have indicated that immune response of the CNS, in which glial cells are critically involved, plays an important role in the development and persistence of neuropathic pain [3, 4]. Hyperalgesia and allodynia are frequently induced by increased levels of proinflammatory cytokines, including TNF-*α*, IL-1*β*, and IL-6, in the CNS, and the resulting or accompanying activation of glial cells [5]. Thus cytokines and glial cells in the CNS have been recognized as powerful modulators of nociception and hold potent potential to the control of neuropathic pain [6].

Puerarin, the main constituent of* Radix Puerariae*, has a variety of pharmacology characteristics [7]. Puerarin has been shown to effectively inhibit proinflammatory cytokine production and/or glia cell activation in a variety of diseases [8–10]. We speculated that puerarin could also be effective in the intrathecal treatment of neuropathic pain via inhibition of spinal inflammation; thus the present study was designed to investigate the effects of puerarin on neuropathic pain using two most commonly used rodent pain models (partial sciatic nerve injury and diabetes). In addition to pain, the effects of puerarin on proinflammatory cytokine production and glial activation in the spinal cord were also examined.

## 2. Materials and Methods

### 2.1. Animals

Male Sprague-Dawley rats (220–250 g) were purchased from Shanghai Experimental Animal Center, Chinese Academy of Sciences (Shanghai, China). The rats were housed in a temperature-controlled room (22–25°C) in plastic cages (5 animals per cage) with a 12-hour light/dark cycle and had free access to food and water. All animal experiments were approved by Xiamen University Animal Care and Use Committee. All efforts were made to minimize the animal suffering and the number of animals used.

### 2.2. Drugs

For intrathecal injections, Puerarin and fluorocitrate were dissolved in artificial cerebrospinal fluid (ACSF). The solution was completed in distilled water. Puerarin and fluorocitrate and all other reagents were purchased from Sigma-Aldrich.

### 2.3. Intrathecal Catheterization

For intrathecal drug administration, intrathecal catheterization [11] was performed by advancing the PE-5 catheter 8.5 cm caudally to the lumbar enlargement through an incision in the cisternal membrane of rats that were under isoflurane mask anesthesia. The catheter was externalized and secured to the musculature at the incision site. Rats showing any neurologic dysfunction, such as paralysis or urine incontinence after catheterization, were euthanized. A proper location of the catheter was confirmed by the hind limb paralysis after 10 *μ*L intrathecal injection of 2% lidocaine (Sigma) 1 day after catheterization. Only rats displaying normal grooming, ambulation, and weight gain after catheterization were used in the following study.

### 2.4. Type 1 Diabetic Neuropathic Pain Model

Diabetes was induced in rats by a single intraperitoneal (i.p.) injection of streptozotocin (STZ, Sigma; 60 mg/kg, i.p.) [12]. Age-matched control rats received an equal volume of vehicle (0.01 M citrate buffer, pH 4.5). Blood was collected via the tail vein at 72 h after STZ injection to measure glucose concentration. Rats with a blood glucose level over 16.67 mmol/L were considered to be diabetic and included in the subsequent experiments. Puerarin (4, 20, and 100 nM), fluorocitrate (1 nM, served as a positive control), or vehicle was administered intrathecally once daily for 7 consecutive days beginning on day 20 after STZ injection. The mechanical withdrawal threshold of the right hind paw was measured before STZ injection (baseline), before drug treatment (predose), and 60 min after each drug administration (postdose) in the morning.

### 2.5. Chronic Constriction Injury (CCI) Model of Neuropathic Pain

The rat CCI model of neuropathic pain was produced according to the method described before [13]. Adult male Sprague-Dawley rats were anaesthetized with chloral hydrate (400 mg/kg, i.p.). The right common sciatic nerve was isolated at mid-thigh level and loosely ligated using chromic gut suture (5-0) at four sites separated by an interval of 1 mm. For sham surgery, the right sciatic nerve was exposed, but the nerve was not ligated. All animals were allowed 3 days to recover from the surgery. Puerarin (4, 20, and 100 nM), fluorocitrate (1 nM), or vehicle was administered intrathecally once daily for 7 consecutive days beginning on day 4 postoperatively. The mechanical withdrawal threshold was measured before surgery (baseline), before drug treatment (predose), and 60 min after each drug administration (postdose) in the morning.

### 2.6. Mechanical Allodynia

Mechanical allodynia was measured using a commercially available electronic von Frey apparatus (Model 2390; IITC Life Science Inc., Woodland Hills, CA) as described previously [14] with minor modifications. Rats were placed into a Plexiglas box on a steel mesh floor. Pressure was applied to the center of the hind paw with the von Frey filament in an upward motion until foot withdrawal. The withdrawal threshold was automatically recorded. The maximum strength of the filament was 55 g. The procedure was repeated after approximately 10 min for each daily session, and the average mechanical withdrawal threshold (MWT) was calculated.

### 2.7. Measurements of NF-*κ*B DNA Binding Activity and Inflammatory Cytokines

NF-*κ*B DNA binding activity measurements were performed using a commercially supplied NF-*κ*B transcription factor binding assay kit (Cayman Chemical, CA, USA) according to the manufacturer's suggested protocol. The assay is based on the principle that only the active form of NF-*κ*B in the sample binds to oligonucleotide containing the NF-*κ*B consensus site (5′-GGGACTTTCC-3′) that is immobilized on the microtiter plate [15]. Briefly, lumbar section of the spinal cord was homogenized in lysis buffer. The nuclear extract was prepared using a nuclear extract kit (Cayman Chemical), and samples of nuclear extract were first incubated overnight at 4°C in wells precoated with a dsDNA sequence corresponding to the NF-*κ*B consensus motif. The NF-*κ*B consensus motif of the assay should bind both human and rat p65. After 5 washes, the samples were incubated overnight at 4°C with primary antibody (rabbit polyclonal, Cayman Chemical) to the p65 subunit of NF-*κ*B. The primary antibody against the p65 subunit of NF-*κ*B used in the assay system is accessible only when NF-*κ*B is activated and bound to its target DNA. Subsequently, samples were incubated for 60 minutes with an HRP-conjugated goat anti-rabbit secondary antibody (Cayman Chemical), followed by colorimetric detection at 450 nm (Multiskan FC, Thermo Scientific). After background subtraction, absorbance measures were referred to a standard curve obtained from a series of duplicate wells containing measured amounts of human recombinant p65 (Cayman Chemical) and then converted to an estimate of the quantity of p65/well, which was normalized by dividing the p65 estimate by the total amount of protein measured in the sample. TNF-*α*, IL-6, and IL-1*β* were quantified using ELISA kits (Abcam, USA) according to the manufacturer's instructions.

### 2.8. Immunohistochemistry

Rats were anesthetized with sodium pentobarbital (50 mg/kg, i.p.), perfused intracardially with 300 mL of 0.9% saline followed by 300 mL of 4% paraformaldehyde in 0.1 M phosphate buffer (PB, pH 7.2–7.4, 4°C). Lumbar spinal segments were removed, postfixed overnight at 4°C, and kept in 30% sucrose in 0.1 M phosphate-buffered saline (PBS) at 4°C. Dissected tissue was mounted in OCT compound and frozen at −20°C. Transverse spinal cord sections (10 *μ*m) were prepared using a cryostat (Microm HM550) and placed in PBS. Sections were washed in 0.01 M PBS twice for 10 min and blocked for 1 h in 5% bovine serum albumin and 0.1% Triton X-100. Free floating tissue sections were incubated overnight at 4°C on a rocker with a rabbit polyclonal antiastroglia marker-GFAP antibody (Abcam; 1 : 1000) or a rabbit polyclonal antimicroglia marker-Iba-1 antibody (Abcam; 1 : 1000). Tissue samples were then washed twice with PBS for 8 min each and incubated with FITC- or Cy3-conjugated anti-rabbit antibody (1 : 300, Jackson Immuno Research Laboratories Inc.) in blocking solution without Triton X-100 for 1 h at room temperature in the dark. Control staining was performed by omitting the primary antibody. Fluorescent images were captured with a digital camera (Olympus). The percentage of positive immunostained area in the dorsal horn was analyzed using Image-Pro software (Plus Version 6).

### 2.9. Western Blot

Nuclear and cytoplasmic protein was extracted with a nuclear and cytoplasmic extract kit (Cayman Chemical). Protein content of each sample was determined using the BioRad protein assay (BioRad Laboratories) according to manufacturer's protocol. For immunoblot analysis, 25 *μ*g of protein was subjected to SDS-PAGE, transferred to a membrane, and probed with specific antibodies: NF-*κ*B p65 (1 : 5000, Abcam); phospho-NF-*κ*B p65 (Ser 536, 1 : 5000, Abcam); Lamin B (1 : 2000, Abcam); *β*-actin (1 : 2000, Abcam). Secondary antibody HRP-conjugated secondary antibodies (Santa Cruz Biotechnology, 1 : 5000) were used to detect binding of antibodies. The membrane was incubated with Clarity Western ECL Substrate (Bio-Rad Laboratories, CA, USA), and the target proteins were then visualized and quantitated using a LAS-3000 luminescent image analyzer (Fujifilm). The results were expressed as a relative ratio of the target protein to reference protein.

### 2.10. Statistical Analysis

The antimechanical allodynia effects were evaluated by the increment of the MWT after drugs treatment and expressed as percentage of maximal possible effect (%MPE): MPE% = [(postdose threshold) − (predose threshold)]/[(baseline threshold) − (predose threshold)] × 100 [16]. It is possible to obtain a negative value of %MPE if the MWT was decreased after treatments, that is, to enhance the allodynic response. Data from the behavior test, ELISA, and immunohistochemistry were analyzed using two-way ANOVA or one-way ANOVA followed by the LSD *t*-test for post hoc analysis. All of the data are presented as mean ± SEM, and all statistical analyses were performed using SPSS software version 16.0 (SPSS Inc., Chicago, IL, USA). A *P* value of <0.05 was considered statistically significant.

## 3. Results

### 3.1. Effects of Puerarin on Pain in Normal Rats

**Figure 1:**
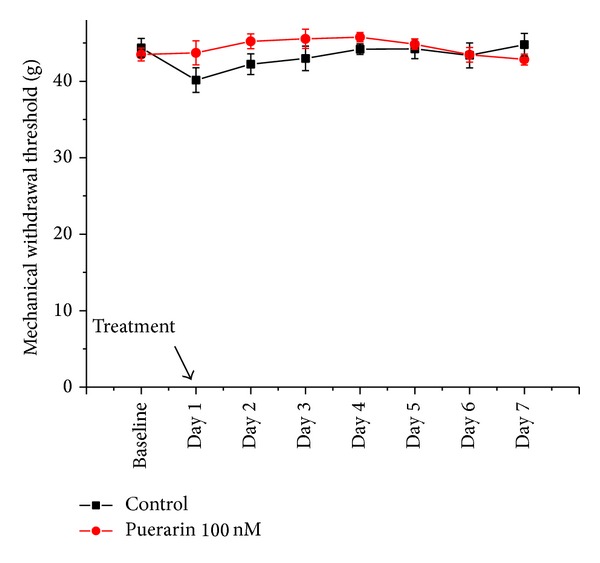
The effects of puerarin on mechanical stimulus test in normal rats. Normal rats received intrathecal 100 nmol puerarin or vehicle. The mechanical withdrawal thresholds (g) of the right hind paws were measured 60 min after drug administration. Data were presented as mean ± SEM. Each group consisted of 5 rats. At 100 nM, puerarin did not affect mechanical withdrawal threshold.

Puerarin (100 nM, i.t., for 7 consecutive days) did not affect MWT (Figure 1).

### 3.2. Effects of Puerarin on CCI- and Diabetes-Induced Neuropathic Pain

**Figure 2:**
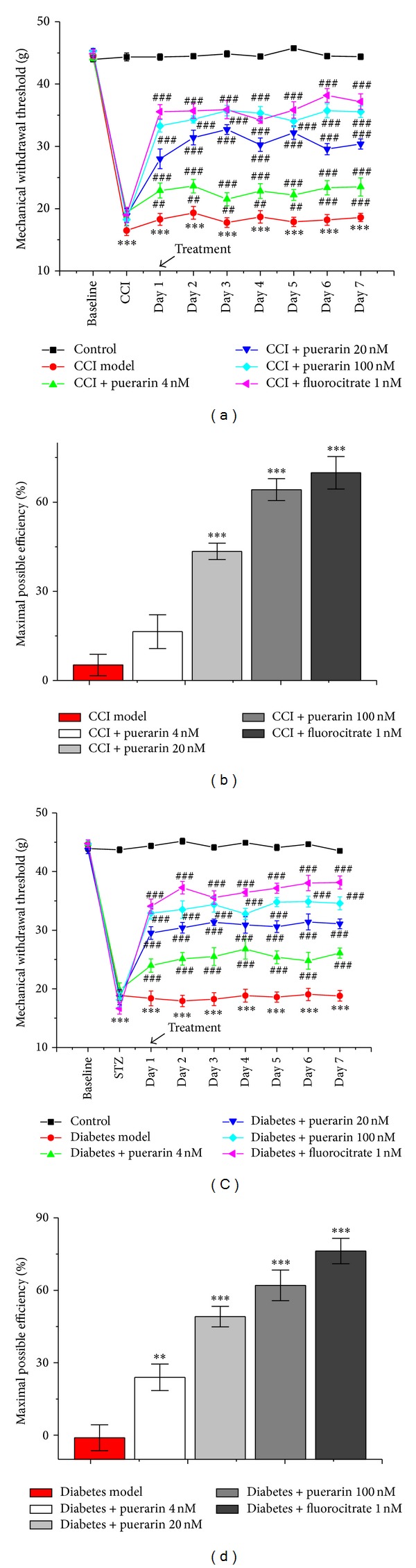
The effects of puerarin on CCI- and diabetes-induced mechanical allodynia. (a), (c) The temporal profile of mechanical withdrawal threshold on CCI- (a) and diabetes- (c) induced mechanical allodynia; (b), (d) the maximal possible efficiency of puerarin on the 11th day after CCI surgery (b) and the 27th day after streptozotocin injection; CCI: chronic constriction injury. Data are presented as mean ± SEM. ***P < 0.001, model group versus control group; ^#^ *P* < 0.05, ^##^ *P* < 0.01, ^###^ *P* < 0.001, puerarin treatment group versus model group (one-way ANOVA with* post hoc* LSD *t*-test). Each group consisted of 6–10 rats.

*Post hoc* tests showed CCI and diabetes significantly decreased (*P* < 0.001, versus sham control) MWT to mechanical stimulation (Figures 2(a) and 2(c), *P* < 0.001, versus sham control), demonstrating the development of mechanical allodynia which persisted for the entire observation period. Comparable to fluorocitrate, puerarin significantly reduced mechanical allodynia in both CCI- and diabetes models compared to the vehicle (Figures 2(a)–2(d)). The MPE of puerarin for CCI- and diabetes-induced neuropathic pain on day 7 was 16.41% ± 5.66% at 4 nM, 43.41% ± 2.75% at 20 nM, and 64.2% ± 3.71% at 100 nM for CCI and 23.93% ± 5.49% at 4 nM, 49.11% ± 4.23% at 20 nM, and 62.07% ± 6.34% at 100 nM for diabetes, respectively (Figures 2(b) and 2(d)). The MPE of fluorocitrate was 69.9% ± 5.48% for CCI and 76.24% ± 5.27% for diabetes, respectively (Figures 2(b) and 2(d)).

### 3.3. Spinal Microglia and Astroglia Activation in CCI and Diabetic Rats

**Figure 3:**
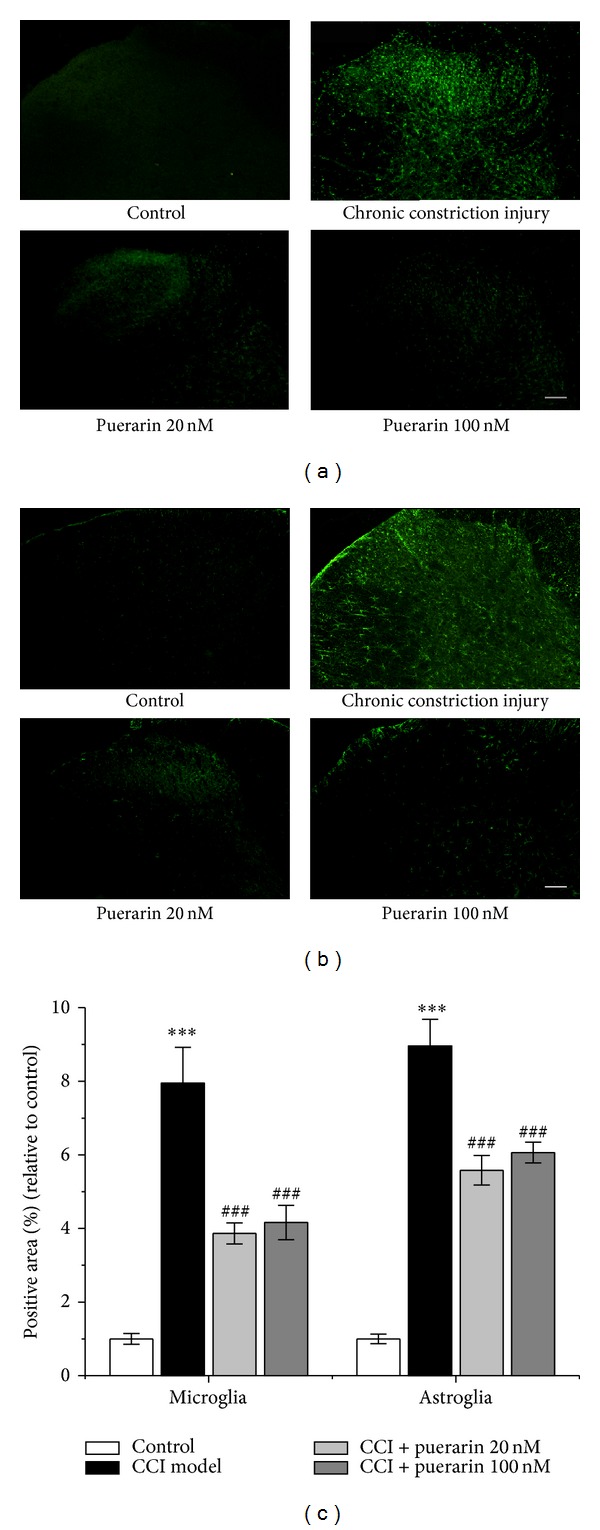
The effects of puerarin on CCI-induced microglia and astroglia activation in ipsilateral spinal dorsal horn of rats. (a) and (b) Representative images of ipsilateral spinal dorsal microglia (a) and astroglia (b) activation in control rats, CCI rats, CCI rats receiving vehicle, 20 and 100 nM puerarin on the 11th day after CCI surgery. Scale bar: 100 *μ*m. (c) Quantification of microglia activation in the spinal cord on the 11th day after CCI surgery. CCI: chronic constriction injury. Data are presented as mean ± SEM. ***P < 0.001, CCI model versus control; ^###^ *P* < 0.001, puerarin versus CCI model (one-way ANOVA with* post hoc* LSD *t*-test). Each group consisted of 6–10 rats.

In CCI groups, staining of the microglia activation marker Iba-1 was barely detectable in the spinal cord in sham control rats (Figure 3(a)). The number of Iba-1 immunoreactive cells significantly increased in the dorsal horn ipsilateral to the CCI injury on day 11 after surgery (Figures 3(a) and 3(c), *P* < 0.001). Puerarin reduced the enhanced Iba-1 immunoreactivity in the spinal cord (Figures 3(a) and 3(c), *P* < 0.001 for puerarin 20, 100 nM). Rats receiving sham CCI surgery showed a low-level staining for GFAP, an astroglia activation marker, in the spinal cord (Figure 3(b)). The number of GFAP immunoreactive cells significantly increased in the dorsal horn ipsilateral to CCI injury on day 11 after surgery (Figures 3(b) and 3(c), *P* < 0.001). Puerarin reduced the increase of GFAP immunoreactivity in the spinal cord caused by CCI (Figures 3(b) and 3(c), *P* < 0.001 for puerarin 20, 100 nM).

**Figure 4:**
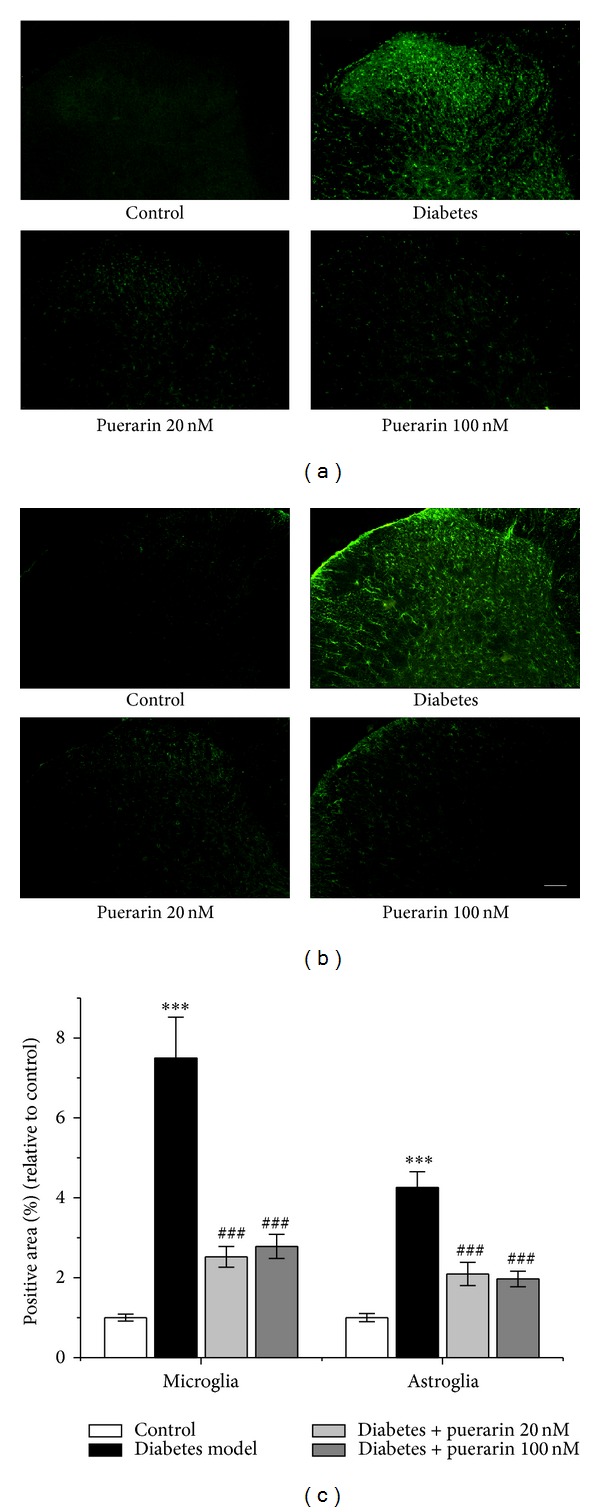
The effects of puerarin on diabetes-induced microglia and astroglia activation in ipsilateral spinal dorsal horn of rats. (a) and (b) Representative images of ipsilateral spinal dorsal microglia (a) and astroglia (b) activation in control rats, diabetic rats receiving vehicle, 20 and 100 nM puerarin on the 27th day after streptozotocin injection. Scale bar: 100 *μ*m. (c) Quantification of microglia and astroglia activation in the spinal cord on the 27th day after streptozotocin injection. Data are presented as mean ± SEM. ***P < 0.001, diabetic rats versus control; ^###^ *P* < 0.001, puerarin versus diabetes model (one-way ANOVA with* post hoc* LSD *t*-test). Each group consisted of 6–10 rats.

As for the diabetic groups, there is a similar trend. A low level and diffuse staining of Iba-1 was noticed in the spinal cord of rats receiving vehicle injection (Figure 4(a)). The number of Iba-1 immunoreactive cells dramatically increased in the dorsal horn on day 27 after diabetes induction (Figures 4(a) and 4(c), *P* < 0.001). Puerarin attenuated the increase of Iba-1 immunoreactivity in the spinal cord in the diabetic rats (Figures 4(a) and 4(c), *P* < 0.001 for puerarin 20, 100 nM). Staining of GFAP in the spinal cord was barely noticeable in rats receiving vehicle (Figure 4(b)), while significantly increased in the dorsal horn on day 27 after diabetes induction (Figures 4(b) and 4(c), *P* < 0.001). Puerarin reversed the elevated GFAP immunoreactivity in the spinal cord (Figures 4(b) and 4(c), *P* < 0.001 for puerarin 20, 100 nM).

### 3.4. Effect of Puerarin on TNF-*α*, IL-1*β*, and IL-6 Production and NF-*κ*B Activation in CCI and Diabetic Rats

**Figure 5:**
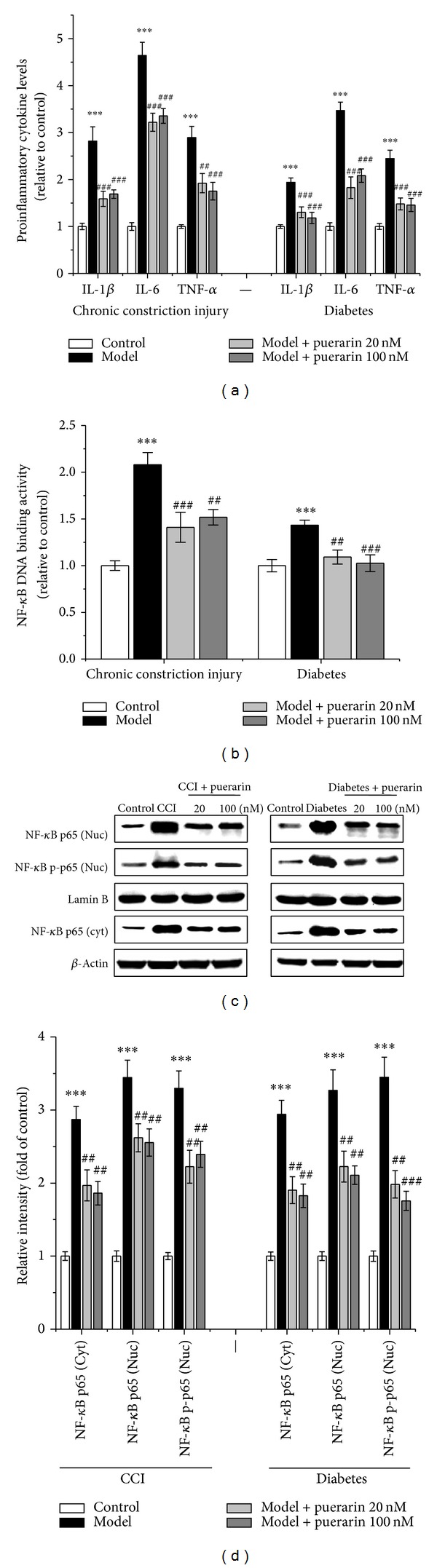
The effects of puerarin on CCI- and diabetes-induced proinflammatory cytokines overexpression (a) and NF-*κ*B overactivation ((b), (c), (d)) on the 11th day after CCI surgery, and on the 27th day after streptozotocin injection in rat spinal cord. Data are presented as mean ± SEM. CCI: chronic constriction injury; Cyt: cytoplasmic; Nuc: nuclear. ***P < 0.001, CCI and diabetes models versus control; ^#^ *P* < 0.05, ^##^ *P* < 0.01, ^###^ *P* < 0.001, puerarin versus CCI and diabetes models (one-way ANOVA with* post hoc* LSD *t*-test). Each group consisted of 6–10 rats.

CCI increased TNF-*α*, IL-1*β*, and IL-6 production and NF-*κ*B activation in the spinal cord (*P* < 0.001 versus control, Figure 5). Puerarin attenuated CCI-induced increase of TNF-*α*, IL-1*β*, and IL-6 (*P* < 0.001 for IL-1*β* and IL-6 at 20, 100 nM, *P* < 0.01 and *P* < 0.001 for TNF-*α* at 20 nM and 100 nM, resp., Figure 5(a)). The elevated NF-*κ*B DNA binding activities were also significantly reduced with puerarin treatment (*P* < 0.001 and *P* < 0.01 at 20 nM and 100 nM, resp., Figure 5(b)). Moreover, the overexpression of NF-*κ*B p65 and p65 nucleus translocation was significantly reduced with puerarin treatment (*P* < 0.01, Figures 5(c) and 5(d)).

With the same pattern, TNF-*α*, IL-1*β*, and IL-6 production and NF-*κ*B activation were significantly elevated in the spinal cord of diabetic rats (*P* < 0.001 versus control, Figure 5). Puerarin at 20 and 100 nM decreased the diabetes-induced elevation of TNF-*α*, IL-1*β*, and IL-6 (*P* < 0.001, Figure 5(a)) and NF-*κ*B DNA binding activities (*P* < 0.01 and *P* < 0.001 at 20 nM and 100 nM, resp., Figure 5(b)). Besides, the overexpression of NF-*κ*B p65 and p65 nucleus translocation was significantly inhibited with puerarin treatment (*P* < 0.01, Figures 5(c) and 5(d)).

## 4. Discussion

Neuropathic pain is caused by aberrant sensory processing in either the peripheral nervous system and/or the central nervous system and is currently lacking efficacious therapy. We found that consecutive intrathecal administration of puerarin to rats inhibited the mechanical and thermal nociceptive response induced by CCI and diabetes, which might acted through inhibiting the activation of microglia and astroglia in the spinal dorsal horn and reducing the upregulated levels of nuclear factor-*κ*B (NF-*κ*B) and proinflammatory cytokines including IL-6, IL-1*β*, and TNF-*α*, in the spinal cord.

Neuropathic pain is characterized by spontaneous pain, increased responsiveness to pain stimuli (hyperalgesia), and pain perceived in response to normally nonnoxious stimuli (allodynia) [1]. A growing body of literature indicates that the enhanced spinal neuroimmune and neuroinflammatory activities initiate and maintain neuropathic pain after the primary nerve injury [6, 17]. Specifically, the proinflammatory cytokines such as TNF-*α*, IL-1*β*, and IL-6 have been strongly implicated in the initiation and development of neuropathic pain after nerve injury [18, 19]. Neuropathic pain is usually accompanied by peripheral and central nervous system damage. Nerve injury leads to a rapid release of pain-related mediators, such as TNF-*α*, IL-1*β*, IL-6, and prostaglandins, resulting in inflammatory responses that sensitize the CNS and facilitate pain processing [20]. NF-*κ*B plays a vital role in these processes and is an important mediator in the regulation of proinflammatory cytokines and inflammatory and immune responses [21]. A number of inflammatory mediators (i.e., TNF-*α*, IL-1*β*, IL-6, NO, and TGF-1*β*) that are implicated in the modulation of the neuropathic pain can activate NF-*κ*B or can be activated by NF-*κ*B [22]. Inhibition of the expression of NF-*κ*B and proinflammatory factors (TNF-*α*, IL-1*β*, and IL-6) can alleviate mechanical allodynia and thermal hyperalgesia in chronic constriction injury (CCI) rat model [23]. Taken together, NF-*κ*B is one of the most important transcription factors regulating gene expression of the proinflammatory cytokines [24, 25] and is implicated in the initiation and development of neuropathic pain via a neuron-mediated way of central sensitization and glia cells-mediated expressions of proinflammatory cytokines and pain mediators [6, 23, 26].

Resident astroglia and microglia in the CNS are known to play important roles in neuroinflammation [27]. Spinal glial activation has been demonstrated in a variety of animal pain models, including models of neuropathic and inflammatory pain [28]. Following stimulation, spinal glia cells proliferate, undergo morphological changes, increase the expression of cell surface receptors, and increase the production and release of proinflammatory cytokines (TNF-*α*, IL-1*β*, and IL-6) and other cytotoxic products, which in turn enhance pain transmission in the dorsal horn of the spinal cord [28, 29]. Also, the spinal neuroimmune and neuroinflammatory activation is a positive feed-forward loop mediated by proinflammatory cytokines, glia cells, and NF-*κ*B [28].

**Figure 6:**
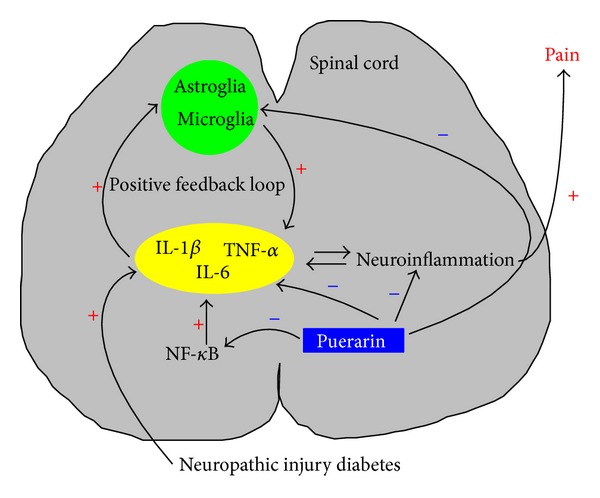
Hypothetical mechanisms of antineuroinflammation activity of puerarin.

In the current study, two classic models for neuropathic pain: a sciatic nerve chronic constriction injury model [30] and a diabetic neuropathy model [31, 32], were used. We showed that fluorocitrate, which specifically blocks glial metabolic activity by inhibiting the activity of aconitase, a Krebs cycle enzyme found exclusively in glia [33–36] and served as positive control in this study, reduced the pain response in both models as previous reported [12, 37, 38]. Consistent with the previous studies [5, 23, 27, 39–42], the expression of TNF-*α*, IL-1*β* IL-6, and NF-*κ*B p65 (p-p65) was dramatically increased in spinal cord, and the spinal astroglia and microglia were drastically activated in both the CCI injury and diabetic models. The effects of puerarin were dose-dependent and comparable to that of fluorocitrate. Puerarin did not affect the pain threshold in normal rats, suggesting the effects of puerarin on neuropathic pain are specific. The fact that puerarin inhibited the overexpression of spinal TNF-*α*, IL-1*β*, IL-6, and NF-*κ*B p65 (p-p65) and spinal glia activation associated with neuropathic pain in our study suggest that the action of puerarin against neuropathic pain involves the regulation of the neuroinflammatory process and the neuroimmune system in general (Figure 6).

## 5. Conclusion

In summary, intrathecal administration of puerarin produces dose-dependent antinociceptive effects in CCI- and diabetes-induced neuropathic pain. One possible mechanism is the inhibition of neuroinflammatory process and glia activation. The present study also suggests that puerarin is a promising platform for developing novel agents for the treatment of neuropathic pain.
